# Teaching Skills Training for Pre-clinical Medical Students Through Weekly Problem-Based Learning Teaching Topic Presentations and Directed Feedback

**DOI:** 10.1007/s40670-023-01912-x

**Published:** 2023-10-18

**Authors:** Gregory Schreck, Dale Netski, Edward Simanton, Rosalie Kalili

**Affiliations:** 1 Department of Internal Medicine, University of Utah, Salt Lake City, Utah, USA; 2 Department of Medical Education, Kirk Kerkorian School of Medicine at UNLV, 625 Shadow Lane, Office 514 , Las Vegas, NV 89106 USA

**Keywords:** Teaching skills, Problem-based learning, Learning issue presentations, Medical students, Pre-clinical

## Abstract

**Problem:**

Medical students commonly encounter scenarios in which they are charged with teaching medical content, but studies find a paucity of teaching skills training especially in the pre-clerkship phase of undergraduate medical programs.

**Intervention:**

Videos lessons were created to instruct on five teaching skills identified as useful for presenting short lessons on medical topics: effective learning objectives, appropriate lesson complexity, audience engagement, relevance to practice, and resource selection. A rubric was generated to assess the performance level of each teaching skill.

**Context:**

First-year medical students viewed the video lessons and were instructed to implement these teaching skills for the creation and delivery of weekly learning issue (LI) presentations within a problem-based learning (PBL) course. PBL facilitators assessed students by using the rubric to assign a score of 0–2 corresponding to the level of skill performance.

**Impact:**

Scores in every dimension of our LI assessment rubric showed significant improvement above week 1 at the end of the initial 4 weeks of practice and assessment. Follow-up assessment showed durable performance and significant improvement for 3 out of 5 at weeks 8 and 12.

**Lessons Learned:**

Our novel framework was effective in fostering the adoption and implementation of five teaching skills among first-year medical students over a 4-week period, with most skills remaining durable over 12 weeks. Furthermore, end-of-course surveys showed that students found feedback received using the framework helpful in improving their LIs, and faculty reported that student LI presentation quality improved overall.

**Supplementary Information:**

The online version contains supplementary material available at 10.1007/s40670-023-01912-x.

## Introduction

In the LCME standards for undergraduate medical education, a curriculum must include instruction in “communication skills as they relate to communication with patients and their families, colleagues, and other health professionals [1].” This standard encompasses many situations in which a student is expected to be a teacher of medical content, such as creating medical topic presentations for class sessions, educating a patient on the disease process, or summarizing landmark papers on rounds. Although medical students commonly encounter scenarios in which they are charged with teaching medical content, studies often find a paucity of teaching skills training in undergraduate medical programs, especially for students in preclinical years.

A survey of US MD programs found that among 99 respondent schools, 44 offered courses in which students were trained and were allowed to practice teaching skills [2]. This investigation found that of these 44 programs, only four required training. Similarly, a review of 19 Australian medical schools found that only five required teaching skills training and practice [3], and a review of 22 British medical schools found 9 had required teaching skills training and practice [4]. Additionally, these courses tend to be delivered to students later in their medical training. A review of 19 published courses on teaching skills training for medical students found that only four offered instruction in teaching skills to students in years 1–2 of school [5]. Together, these studies suggest that while training related to teaching skills is offered to medical students, they were often not required and completed later in subsequent training phases. It appears to be the norm that teaching skills training is treated as an optional skillset to be learned and practiced by students who opt-in during an elective block or pathway. This stands in contrast to the LCME standard and expectation that all students and physicians regularly act as communicators and teachers of medical content. This discrepancy prompted the authors to explore opportunities to offer basic teaching skills training for all students as part of the core curriculum by integrating it into our institution’s preclinical phase, early on in the curriculum.

Our institution’s problem-based learning (PBL) course was identified as such an opportunity to allow for integration of teaching skills training and practice into an existing element of the curriculum. PBL is a standardized framework for case-based small-group learning, and it already offers students opportunities to teach their peers medical content through learning issues (LIs). LIs are a core component of standard PBL frameworks and require that every student select a topic during case review and teach that topic to their peers at a future group session. Though the PBL framework includes this opportunity for students to teach each other, it does not specify how students should plan, construct, or teach their lessons to their peer groups [16]. This gap in the standard PBL curricula mirrors the above discrepancy in which students are expected to teach, but are not explicitly trained in teaching skills. This led to the identification of PBL as an opportunity for direct instruction on teaching skills and the development of a novel framework for teaching skills training which was implemented within our institution’s existing PBL course. The purpose of this study is to assess the impact of this novel framework that offers teaching skills training to all preclinical medical students within an existing element of our institution's core curriculum. This framework represents a novel contribution to the literature in terms of the timing of implementation and structured instructional approaches.

## Materials and Methods

### Target Learners and Classroom Environment

To instruct preclinical medical students in select teaching skills, we developed a framework that utilizes video lessons and an aligned rubric for performance assessment and targeted feedback. We identified problem-based learning (PBL) tutorial sessions as the classroom environment to implement the teaching skills framework because it is one of the learning methods used at the Kirk Kerkorian School of Medicine during the first 18 months designated as phase 1, preclinical training time. PBL tutorial sessions are aligned with organ system–based courses and are scheduled regularly — twice a week for 3 hours each session. Each PBL group comprised a single facilitator with 7 to 8 medical students in each group. Students are required to create and teach a Learning Issue (LI) presentation (a short lesson on a medical topic for their peers) once every week, about which faculty and group members are encouraged to give feedback to help each student improve their future LI presentations. Altogether, this weekly cycle of lesson creation, delivery, and feedback was identified as an ideal opportunity for implementing structured and targeted development of teaching skills.

### Teaching Skills Selection

We identified five teaching skills that complement the components of LI presentations and proved useful for the creation and delivery of these short lessons on medical topics. Each teaching skill was taught to students in a short video lesson and had its own dimension and performance targets in an LI assessment rubric. The lessons and rubric performance targets were adapted from teaching practices characterized by several widely used educational theories. The teaching skills selected were (1) creating effective learning objectives, (2) targeting lesson complexity to the level of the learner, (3) actively engaging learners, (4) connecting lessons to real-world examples, and (5) choosing appropriate and accessible resources for lesson creation. Our lesson and rubric dimension on creating effective learning objectives were adapted from the SMART learning objectives framework [6], with a focus on specific and achievable elements. Our lesson and rubric dimension on targeting lesson complexity to the level of the learner introduced students to the theory of the zone of proximal development [7] as well as cognitive load theory [8]. Our lesson and rubric dimension on engaging the learner referenced Webb’s depth of knowledge levels [9] as a tool to create rigorous opportunities for audience engagement. Our lesson and rubric dimension on establishing relevance to real-world practice referenced transfer theory and highlighted the importance of drawing parallels between lessons and real-world applications [10, 12]. Finally, our lesson and rubric dimension on resource selection referenced the Ebbinghaus forgetting curve to highlight the importance of resources are able to be revisited by the learner [13]. We reviewed additional literature to inform adaptation of these teaching skills to the medical education context [10, 11, 14, 15]. We also conferred with the PBL facilitators the specific components of an LI presentation that typically provided feedback to inform the scope of our teaching skill selection.

### Video Mini-series and Aligned LI Rubric Creation

We created a video mini-series that consisted of five video lessons to introduce and instruct the students on each of the five teaching skills (Appendix A). Each lesson offered a brief theoretical explanation of the specified teaching skill, an explanation of how to implement the teaching skill within an LI presentation, and a closing activity for practicing the skill. The video lessons ranged from 5 to 18 minutes in length and were indefinitely available to the students. We created an accompanying LI rubric with five dimensions, with each dimension representing one of the five teaching skills, and three performance levels for each dimension (Appendix B). The three performance levels were represented as follows: 0, teaching skill not performed; 1, teaching skill performed partially; and 2, teaching skill performed completely. The number scoring assigned to each performance level (0, 1, or 2) were selected for ease of use. A performance target level of 2 for each teaching skill aligned with the learning objectives of each video lesson, with a goal total rubric score of 10.

### Structured Instructional Approach

First-year medical students at the Kirk Kerkorian School of Medicine at UNLV received training in PBL-related tasks during the time designated for orientation, leading up to the start of the classes. We offer training on concepts such as small-group learning, LI creation, and feedback. During the training session related to LI creation, we assigned the five video mini-series as mandatory viewing to the Class of the 2025 cohort (*n* = 62, 27 males, 35 females). During the training session related to feedback, we introduced the LI rubric and performance goals for each of the five teaching skills. Students then began the PBL course and were instructed to implement the five teaching skills when creating and delivering weekly LI presentations. The students were informed that the LI rubric would be used by the facilitator to assess each LI presentation and that the score was formative, with the purpose of providing immediate and specific feedback for teaching skill target performance improvement. The students had continuous access to video lessons and the accompanying rubric for reference. We encouraged students to use the rubric to score their groupmates’ LI presentations in order to familiarize themselves with the teaching skills performance targets.

### PBL Facilitator Training

The faculty at our institution that serve as PBL group facilitators have a range of experience in leading PBL tutorial sessions between 2 and 4 years. PBL facilitators typically receive faculty development and training sessions 2–3 times a year as a group on topics such as small group facilitation, active learning methodologies, or how to give skills-based feedback. We introduced the video lesson series and the aligned rubrics during regularly scheduled summer training sessions. We instructed the facilitators to watch the videos and review the rubric beforehand. Then, at the assigned training session, we described how to use the LI rubric and help students attain their performance targets. We informed the facilitators that for the first 4 weeks of the course, they were required to use the LI rubric to score every student LI presentation and deliver immediate formative feedback on each of the five dimensions scored. The rational is threefold: (1) to emphasize that each teaching skill is expected to be practiced and performed every week, (2) to offer explanations and directions should students have questions about the teaching skills target, and (3) to provide timely and specific feedback directed at teaching skill performance improvement. After this initial four week period, facilitators were no longer required to score every LI and give immediate feedback on every rubric dimension. Rather, students were encouraged to continue using the rubrics and video lessons as a reference for performance targets during LI presentations, and instructors were encouraged to give as needed feedback on specific dimensions at their discretion. Requirements were relaxed in this manner in order to assess what level of skill durability might be observed without imposing the time intensive assessment and feedback requirements indefinitely. Scoring of every LI was then only done at weeks 8 and 12, the timing of which corresponded to mid-course and end course, to assess skill durability. These scores were collected without requiring that faculty deliver immediate comprehensive feedback as in weeks 1–4. Additionally, faculty were instructed to remind students that all feedback related to the rubric scores was solely formative, intended to improve LI presentation quality, and not impact their PBL course grade. Two weeks after implementing the rubric, we had a follow-up meeting with the facilitators to provide an opportunity for them to share their experience using the rubric, ask questions, and make suggestions on the future use or iteration of the rubric.

### Assessing Impact of the Teaching Skills Framework

We used a mixed methods design to assess the impact of our novel teaching skills framework. Quantitative data consisted of LI rubric scores and a Likert scale survey of student attitudes regarding the novel framework. Qualitative data consisted of facilitators’ attitudes, which were collected by a survey.

We instructed the faculty to turn in the completed rubrics weekly for the first 4 weeks of the PBL course so that the scores could be assessed for teaching skill uptake (Table 1). We instructed faculty to use and complete unannounced rubrics at the end of two consecutive 4-week intervals — weeks 8 and 12. Scores of which were to assess and represent the durability of teaching skills performance (Table 1). We used Google sheets to obtain the tally rubric scores and performed data analysis. We calculated the rubric scores to obtain an average total score by week, as well as the average scores for each of the five individual rubric dimensions. We performed independent *t*-tests to assess significant elevation in average scores, as well as each individual dimension, at weeks 4, 8, and 12. We administered a 5-item survey to assess student reactions to the video mini-series and LI rubric in week 7 of their first PBL course (Table 2). We asked the PBL faculty to complete a survey to assess their reactions to the content and perceived impact of the video lessons and the LI rubric (Table 3). This project was approved for Category 1 exemption by the UNLV School of Medicine Institutional Review Board (1800301–1).

**Table 1:** Mean LI rubric scores for PBL tutorial session weeks 1–4, 8, and 12

**Mean score**						
**Week (*n*)**	**Total**	**Learning objective**	**Complexity**	**Engagement**	**Relevance**	**Resources**
1 (*n* = 62)	7.26 (1.66)	1.06 (0.74)	1.53 (0.59)	1.39 (0.64)	1.53 (0.67)	1.74 (0.57)
2 (*n* = 62)	8.73** (1.27)	1.79** (0.41)	1.82* (0.39)	1.69* (0.46)	1.68 (0.54)	1.74 (0.48)
3 (*n* = 61)	9.00** (1.20)	1.82** (0.39)	1.75* (0.43)	1.67* (0.51)	1.84* (0.42)	1.92* (0.28)
4 (*n* = 53)	9.32** (0.89)	1.98** (0.14)	1.92** (0.27)	1.70* (0.54)	1.79* (0.41)	1.92* (0.27)
8 (*n* = 57)	8.77** (1.00)	1.81** (0.40)	1.81** (0.40)	1.70* (0.46)	1.68 (0.54)	1.77 (0.42)
12 (*n* = 59)	8.96** (1.11)	1.88** (0.39)	1.91** (0.30)	1.72* (0.60)	1.67 (0.58)	1.79 (0.48)

*LI* learning issues, *PBL* problem-based learning. LI rubric scale 0–2:0, teaching skill not performed; 1, teaching skill partially performed; and 2, teaching skill completely performed.

(#) represents the standard deviation.

** denotes a statistically significant elevation above week 1 score at *p* < 0.0001.

* denotes a statistically significant elevation above week 1 score at *p* < 0.05.

**Table 2:** Student survey response about video mini-series and LI rubric (*n* = 61)

**Item**	**Average score**	**Strongly agree (5)**	**Agree (4)**	**Not sure (3)**	**Disagree (2)**	**Strongly disagree (1)**
I felt the LI mini-series topics were understandable at my learning level	4.2	31%	56%	10%	2%	2%
I felt equipped to start creating my own LIs after completing the LI video mini-series	3.5	10%	43%	31%	16%	0%
I felt that feedback provided from using the LI formative assessment rubric was helpful in improving the format and/or quality of my LIs	4	16%	67%	13%	3%	0%
I would recommend that the LI video mini-series be used for future PBL orientation and training sessions	3.5	13%	41%	33%	10%	3%
I am likely to refer to the LI video mini-series and/or rubric when creating LIs in the future	3.2	11%	31%	30%	20%	8%

*LI* learning issue.

**Table 3:** Comments from PBL facilitators on perceived utility of each teaching skill

**Teaching skill**	**Comments from open response sections**
Learning objectives	“This really sets the stage for the LI and makes it professional and focused” “When given feedback on the objective being achievable they understand how to come up with concise topics”
Complexity	“Often, student teachers will over or underestimate the degree of complexity that can be both taught and understood in the short time appropriate for each LI. Providing this feedback aids learners in creating LIs that are understandable in the time available”
Engagement	“I firmly believe this helps students learn, retain, and have a more enjoyable LI experience” “Highlighting engagement trains student teachers to actively engage their audience rather than fall back on simply lecturing”
Relevance	“It helps keep the student on target, helps relate the LIs to the case and that helps their learning”
Resources	“Resource selection may be the most important area for LI feedback. This learning objective calls the students to the quality and reliability of their sources and sets them up to be successful lifetime learners who rely on valid resources”

*PBL* problem-based learning, *LI* learning issue.

## Results

### Impact of Teaching Skill Framework on Student LI Presentation Scores

We collected and analyzed LI rubric scores from the Class of the 2025 cohort over a 12-week period, (represented in Table 1). The sample size varied by week based on student attendance.

### Survey Results

Sixty-one of the 62-student cohorts completed a survey, representing a 98% response rate, to evaluate the training provided on PBL-related tasks during orientation. The survey included five measurement items related to the video mini-series and LI rubric using a 5-point Likert scale (Table 2).

An optional survey was administered to all the 11 PBL facilitators to assess the utility of the LI rubric. Three completed surveys represented a response rate of 27%. The comments related to the perceived utility of each teaching skill are listed in Table 3.

## Discussion

### Summary

Our study aimed to assess a novel framework that was developed to afford teaching skills training to all preclinical students in a longitudinal manner within our pre-existing institutional curriculum. LIs within PBL were identified as an opportunity to use a widely accepted and well-characterized curricular element to introduce structured learning and practice of teaching skills. Our data showed that existing PBL curricula can serve as a platform to facilitate learning of five selected teaching skills over a 4-week period, and our data suggested that most of these skills will remain durable at 12 weeks.

This was accomplished through video mini-lessons and an aligned formative assessment tool. Students learned, implemented, and received ongoing formative assessment on five specific teaching skills: (1) creating effective learning objectives, (2) targeting lesson complexity to the level of the learner, (3) actively engaging learners, (4) connecting lessons to real-world examples, and (5) choosing appropriate and accessible resources for lesson creation. Our data showed that the video lessons and learning issue (LI) rubric were impactful, as students significantly improved in all five of the teaching skills by the end of a 4-week period of practice and feedback. These teaching skills proved to be durable in weeks 8 and 12. Week 8 and 12 data demonstrated that students continued to perform three of the five teaching skills, with significant improvement from week 1. Survey data demonstrated overall favorable attitudes toward the video mini-series and LI rubric from both students and PBL faculty. These data suggest that we were successful in addressing the perceived need for teaching skills development in medical schools by implementing a structured framework with preclinical medical students through our existing PBL curriculum.

### Reflections

Although week 4 LI rubric scores showed significant improvement in all five teaching skill dimensions, two (resources and relevance) did not at weeks 8 and 12. There are a few possible explanations for this finding. First, the video lessons for relevance and resources were combined, whereas the other teaching skills had their own dedicated video lessons. Second, these two teaching skills were already the highest scored at week 1, with a mean score for resource of 1.74 and relevance of 1.53, with mean scores ranging from 1.07 to 1.74. The students demonstrated that these teaching skills could already be performed close to the target level without practice or feedback. Third, PBL facilitators commented during a training session that the most common reason students would not attain a score of 2 in the resources dimension was because of a preference for and citation of only board study materials over other expected resources. Though this was not qualitatively assessed, it may have impacted overall scores. For the relevance dimension, PBL facilitators suggested during a training session that they felt many students simply forgot to address the relevance dimension in their LI presentations. Again, this was not qualitatively assessed, but students forgetting to address this dimension more often than the others may have driven the impact on scores.

Student survey data from Table 2 reflected largely positive attitudes toward the structured framework, where 87% of students felt that they understood the teaching skills covered in the video mini-series and 83% felt that feedback using the LI rubric was helpful in the creation and delivery of LI presentations. Taken together, these data suggest that our strategy for fostering teaching skill development during the preclinical phase of the curriculum was perceived as approachable and useful by first-year medical students. Qualitative faculty surveys were also positive, with multiple faculty members stating that student implementation of these teaching skills led to various positive changes in LI quality compared with LIs from previous years.

### Lessons Learned

Overall, the structured framework for introducing teaching skills to preclinical medical students was easily implemented and well-received. The use of the video lessons and LI rubric could be seamlessly integrated into any PBL program that uses weekly LIs. The electronic distribution of the video mini-series keeps the resource cost low and the time cost for students is relatively low. Time will be needed for the faculty to watch the entire video mini-series so that a common language for referencing teaching skills and LI rubric performance standards can be established. However, no more than half an hour was used from our regularly scheduled faculty development and training meeting to give instructions on how and when to use the LI rubric or answer any related questions. A follow-up meeting with the faculty did not reveal any difficulties in scoring, clarifying questions, or making suggestions for changes to the LI rubric.

### Limitations

It is important that faculty members who serve as PBL facilitators watch the video mini-series, have a uniform understanding of the five teaching strategies, and correctly use the LI rubric. It is also important that faculty members are experienced in delivering skill-based feedback. If faculty members are not given dedicated time to watch the videos, discuss their understanding of the teaching skills targets, or receive training on feedback, then these could serve as potential challenges. Therefore, it is possible that any of our PBL facilitators’ understandings of the video lesson content and LI rubric scoring could produce interobserver variability. Additionally, some of our PBL facilitators already had personalized frameworks used to assess students and provide feedback on their LI presentation skills. Thus, it is necessary to request uniform adoption and practice of our specific structured framework. It is also noteworthy that our qualitative faculty survey was returned by only three of the 11 facilitators, representing a 27% response rate. Therefore, it is possible that the positive feedback we received regarding the perceived impact of our lessons and rubric was not representative of the opinions of all PBL faculty members using the framework but rather represented the opinions of a few motivated to respond by a strong positive bias.

### Future Steps

Overall, we observed a positive impact on student performance in these teaching skills through LI presentations. Furthermore, the responses from students and staff toward the videos and rubrics were largely positive. We achieved our ultimate goal of helping students learn teaching skills during the preclinical phase of the curriculum. Our hope is that these skills may be carried forward in their careers to make them better teachers and communicators with various audiences, such as colleagues or other healthcare trainees, and especially patients. In the future, it will be necessary to assess the impact of students’ ability to transfer these teaching skills to other teaching tasks required during the clinical phase of medical school training and beyond. The impact of preclinical teaching skills training on a student’s actual capabilities in the clinical context can be used to further assess the value of offering such training early.

### Electronic supplementary material

Below is the link to the electronic supplementary material.

Supplementary file1 (PDF 148 KB)

Supplementary file2 (PDF 300 KB)

Supplementary file3 (PDF 199 KB)

Supplementary file4 (PDF 190 KB)

Supplementary file5 (PDF 181 KB)

## Data Availability

The authors confirm that all data generated or analyzed during this study are included in this published article and its supplementary materials.

## Appendix A: Transcript of Video mini-series

(see supplemental information attachments)

Video Transcript 1 – Introduction.

Video Transcript 2 – Learning objectives.

Video Transcript 3 – Complexity.

Video Transcript 4 – Engagement.

Video Transcript 5 – Relevance and Resources.
