# Supplementary material for: Teaching Skills Training for Pre-clinical Medical Students Through Weekly Problem-Based Learning Teaching Topic Presentations and Directed Feedback
Source: Med Sci Educ. 2023 Oct 18;33(6):1473–80. doi: 10.1007/s40670-023-01912-x (PMC10767181; doi:10.1007/s40670-023-01912-x)
Supplement: Supplementary file 1 — Supplementary file1 (PDF 148 KB) [file 40670_2023_1912_MOESM1_ESM.pdf]

KIRK KERKORIAN SCHOOL OF MEDICINE | UNLV

# Teaching Strategies for Effective LIs

Greg Schreck M.D. M.Ed. Rosalie Kalili M.D.

# Is Teaching a Clinical Skill?

# Is Teaching a Clinical Skill?

- Explaining diseases and treatments to patients
- Presenting medical topics on rounds
- Presenting patients on rounds
- Answering questions from family and friends
- Working with other healthcare providers

# How can we become better teachers?

# Teaching Strategies for Effective LIs:

- **Learning objective** is specific and achievable
- **Complexity** is tailored to the audience
- **Engaging** techniques are be employed
- **Relevance** to the PBL case is explicitly stated
- **Resources** used are reliable and available

# How do we make the most of our practice?

# Course Overview

- Watch the video lessons on 5 Teaching Strategies
- Practice weekly by incorporating the teaching strategies into LIs
- Weekly feedback  and improvement using the formative assessment rubric

# What is the origin of the word "doctor"?
