# Supplementary material for: Teaching Skills Training for Pre-clinical Medical Students Through Weekly Problem-Based Learning Teaching Topic Presentations and Directed Feedback
Source: Med Sci Educ. 2023 Oct 18;33(6):1473–80. doi: 10.1007/s40670-023-01912-x (PMC10767181; doi:10.1007/s40670-023-01912-x)
Supplement: Supplementary file 2 — Supplementary file2 (PDF 300 KB) [file 40670_2023_1912_MOESM2_ESM.pdf]

KIRK KERKORIAN SCHOOL OF MEDICINE | UNLV

Teaching Strategies for Effective LIs

❖ Learning Objectives

Greg Schreck M.D. M.Ed. Rosalie Kalili M.D.

A **broad** LI topic

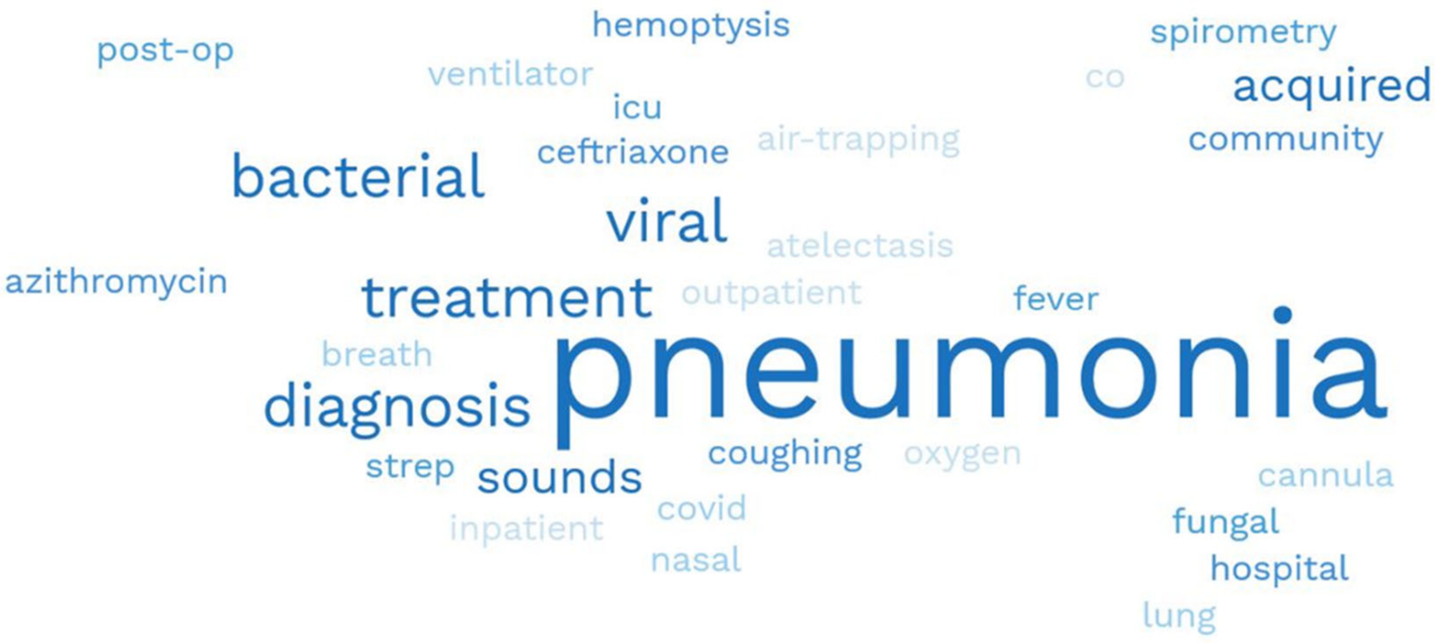

- **Learning objective:**
  - We will **define** what a “learning objective” is and **describe** how to create an effective learning objective

What is a Learning Objective?

- A well-written learning objective outlines the knowledge, skills, and/or attitude the learners will gain from the educational activity and does so in a measurable way

What is a Learning Objective?

Make it specific

- Use the phrase **"We will…."** along with a **verb/verbs** to set a specific objective for your LI

    - **LI:** "Pneumonia"

Make it specific

- Use the phrase **“We will….”** along with a **verb/verbs** to set a specific objective for your LI
    - **LI:** “Pneumonia”
    - **learning objective:** “**We will define** pneumonia and **describe** the clinical manifestation of a patient with community acquired pneumonia”

Make it specific

Make it specific

- Use the phrase **"We will…."** along with a **verb/verbs** to set a specific objective for your LI

    - **LI:** "Pneumonia"
    - **learning objective:** "**We will list** the risk factors for hospital acquired pneumonia (HAP) and **identify** the risk factors for HAP in our patient's case"

**Recall**	**Examine**	**Apply**	**Analyze**	**Evaluate**	**Create**
**Describe**	**Explain**	**Complete**	**Compare/ Contrast**	**Justify**	**Plan**
**Find**	**Compare**	**Use**	**Explain**	**Assess**	**Invent**
**List**	**Discuss**	**Examine**	**Identify**	**Prioritize**	**Compose**
**Relate**	**Predict**	**Illustrate**	**Categorize**	**Recommend**	**Design**
**Write**	**Outline**	**Classify**	**Investigate**	**Inspect**	**Synthesize**

Make it achievable

- The learning objective must be achievable within a reasonable time frame

    - **LI:** “Pneumonia”

Make it achievable

- The learning objective must be achievable within a reasonable time frame

    - **LI:** “Pneumonia”
    - **learning objective:** “**We will review** the presentation, pathophysiology, and treatments, of viral, bacterial, and fungal pneumonia.

Make it achievable

- The learning objective must be achievable within a reasonable time frame

  - **LI:** "Pneumonia"
  - **learning objective:** "**We will compare and contrast** the clinical presentations of viral vs bacterial pneumonia, and **diagram** the classic distribution of each type on a chest x-ray"

- **Learning objective:**
    - We will **define** what a “learning objective” is and **describe** how to create an effective learning objective

- **Performance Target**

	0	1	2
**learning objective**	No learning objective included	learning objective uses a verb/verbs to define a specific objective, OR is achievable in an LI format	learning objective uses a verb/verbs to define a specific objective, AND is achievable in an LI format

Practice: Create **specific** and **achievable** learning objectives from the LI topics below:

- LI 1: “NSAIDs”
- LI 2: “Illness script: “Neonatal Toxoplasmosis”
- LI 3: “How does B12 deficiency cause SCD?”
- LI 4: “Why do humans need vitamin D?”

- **Possible** answers:
    - **LI 1:** “NSAIDs”
    - **learning objective:** We will **diagram** the biochemical pathway by which NSAIDs reduce inflammation
    - **OR**
    - **learning objective:** We will **review** the therapeutic effects of NSAIDs and **review** the common side effects of NSAIDs

- **Possible** answers:
  - **LI 2:** "Illness script: Neonatal Toxoplasmosis"
  - **learning objective:** We will **describe** the clinical presentation of neonatal toxoplasmosis and **review** common treatment options
  - **OR**
  - **learning objective:** We will **compare and contrast** neonatal toxoplasmosis with rubella and CMV

- **Possible** answers:
    - **LI 3:** “How does B12 deficiency cause SCD?”
    - **learning objective:** We will **explain** how B12 deficiency causes nerve damage via methylmalonic acid
    - **OR**
    - **learning objective:** We will **diagram** how B12 deficiency causes nerve damage via methylmalonic acid and **identify** signs of SCD found in our patient

- **Possible** answers:
    - **LI 4:** “Why do humans need vitamin D?”
    - **learning objective:** We will **identify** and **review** biochemical pathways in humans that require vitamin D to function properly
      OR
    - **learning objective:** We will **review** the pathophysiology and clinical presentation of rickets
