# Supplementary material for: Teaching Skills Training for Pre-clinical Medical Students Through Weekly Problem-Based Learning Teaching Topic Presentations and Directed Feedback
Source: Med Sci Educ. 2023 Oct 18;33(6):1473–80. doi: 10.1007/s40670-023-01912-x (PMC10767181; doi:10.1007/s40670-023-01912-x)
Supplement: Supplementary file 3 — Supplementary file3 (PDF 199 KB) [file 40670_2023_1912_MOESM3_ESM.pdf]

KIRK KERKORIAN
SCHOOL OF MEDICINE | UNLV

Teaching Strategies for Effective LIs

❖ Complexity

Greg Schreck M.D. M.Ed. Rosalie Kalili M.D.

A new diagnosis

- **Learning objective:**
    - We will **define** the “zone of proximal development,” and **explain** how it is useful in choosing a level of complexity for our LIs

How complex should we make our LIs?

Building on Prior Knowledge

Building on Prior Knowledge

How do we build on prior knowledge?

Zone of Proximal Development (ZPD)

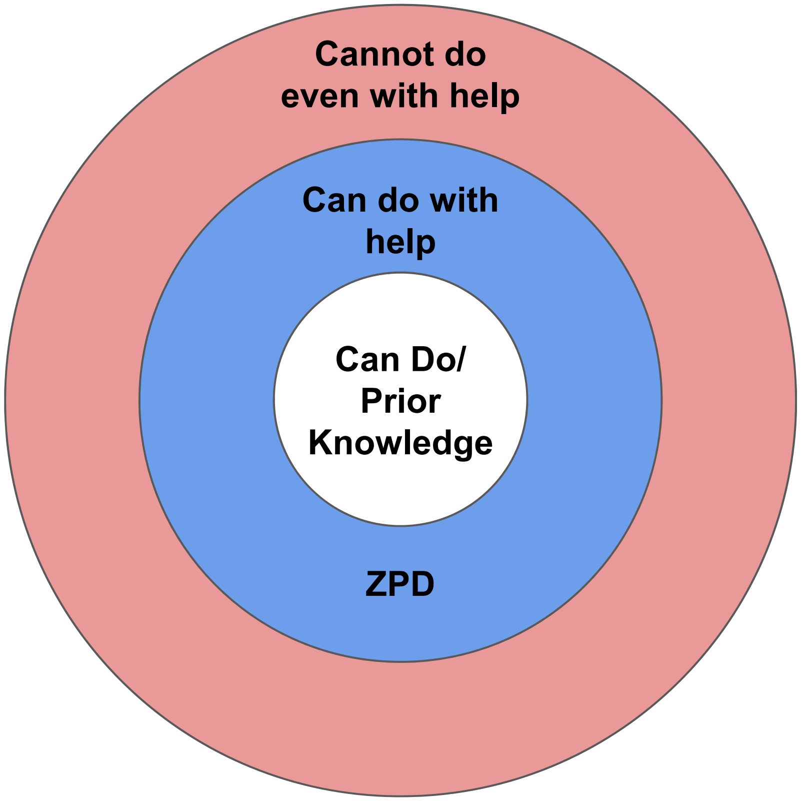

LIs and the ZPD

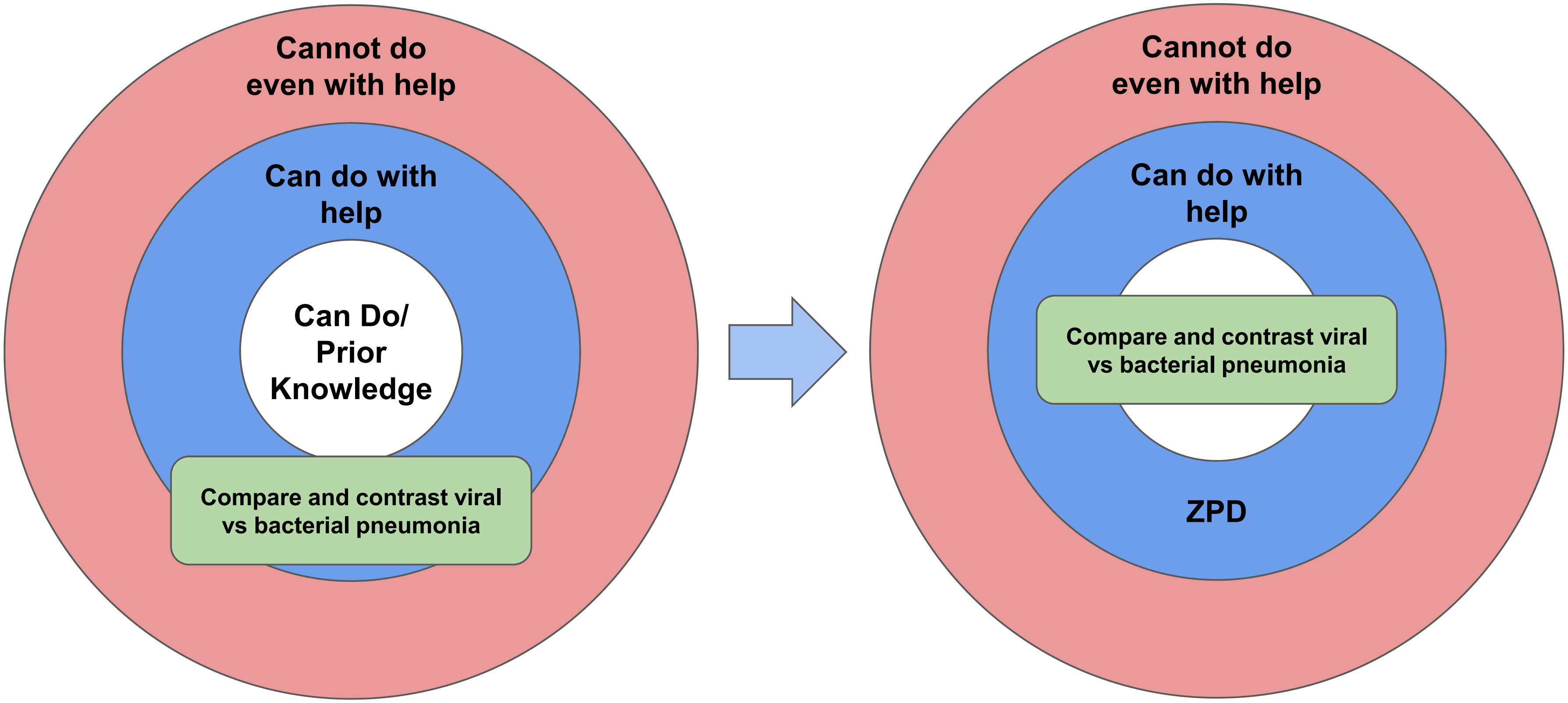

LIs and the ZPD

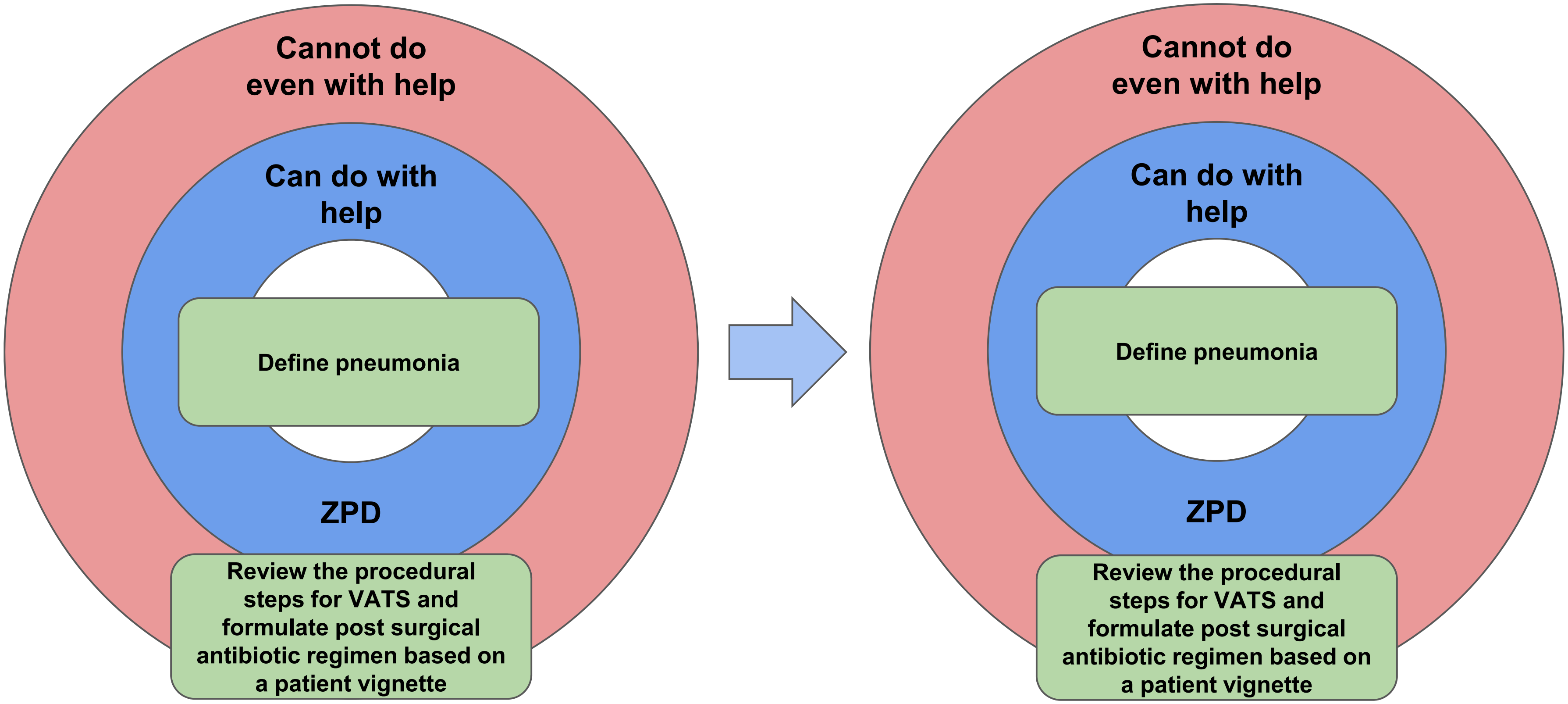

So how do we know where the ZPD is?

Assessments & context clues

Assessments of prior knowledge

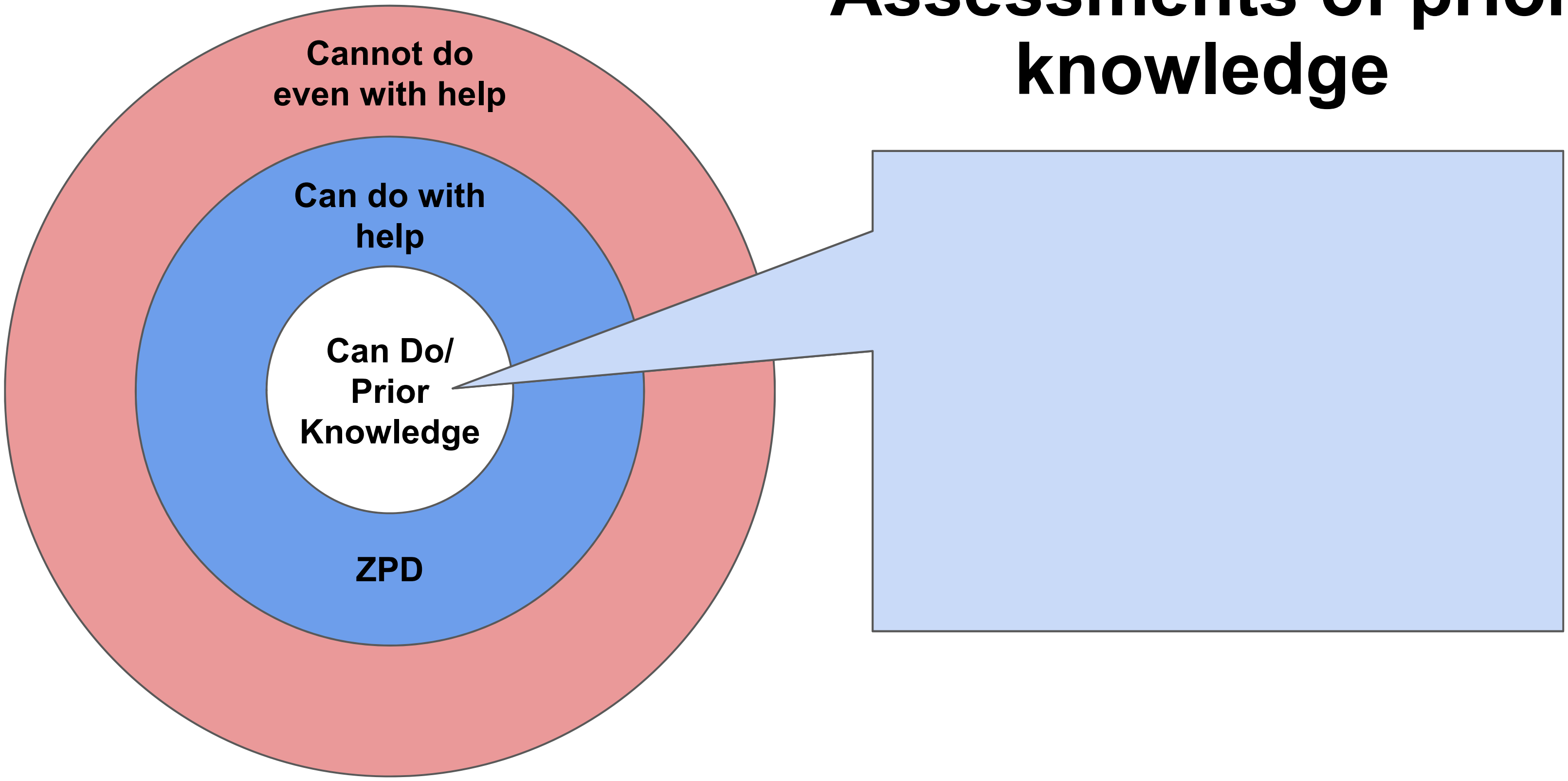

Context clues on prior knowledge

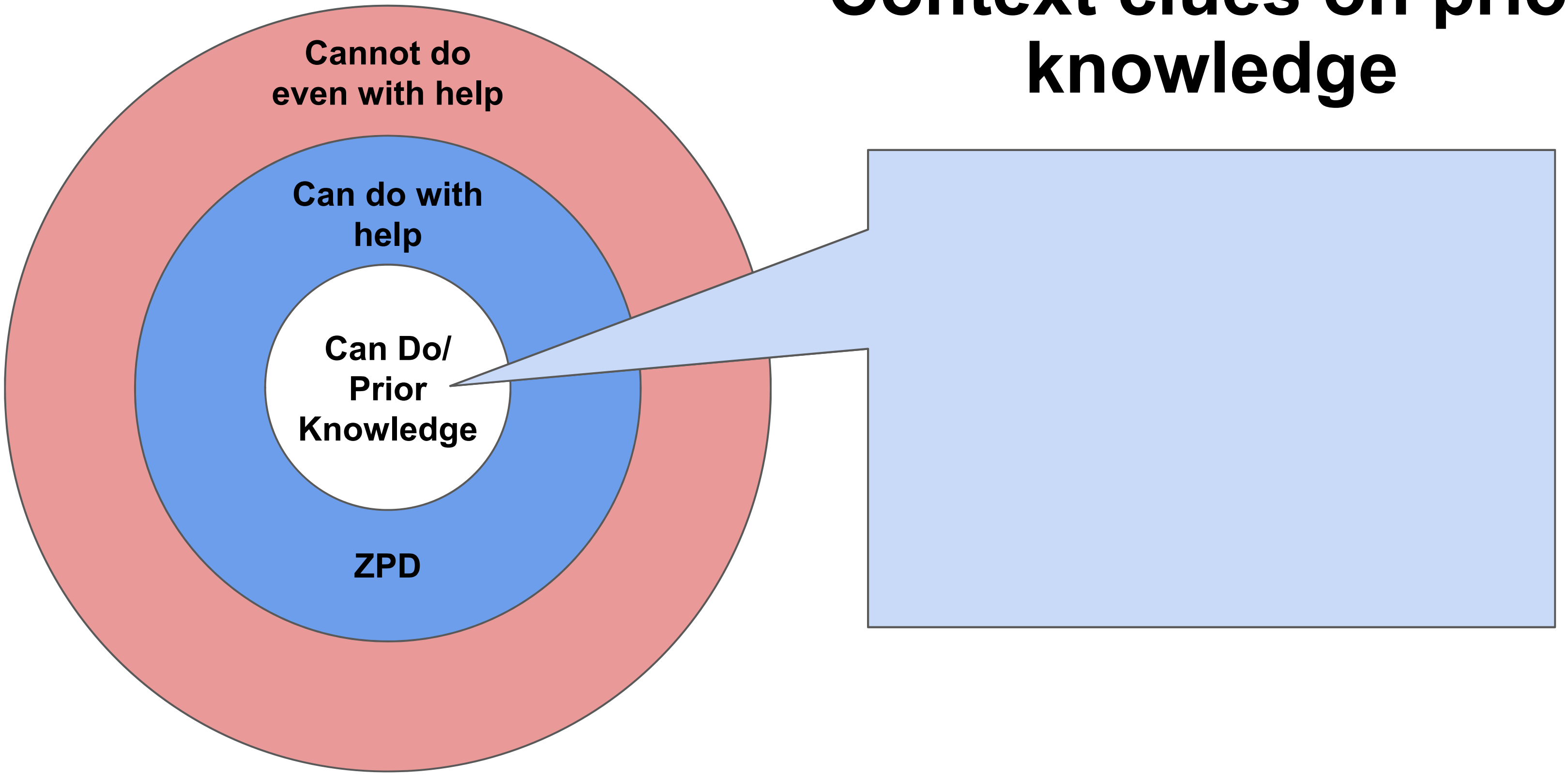

Tailoring Complexity

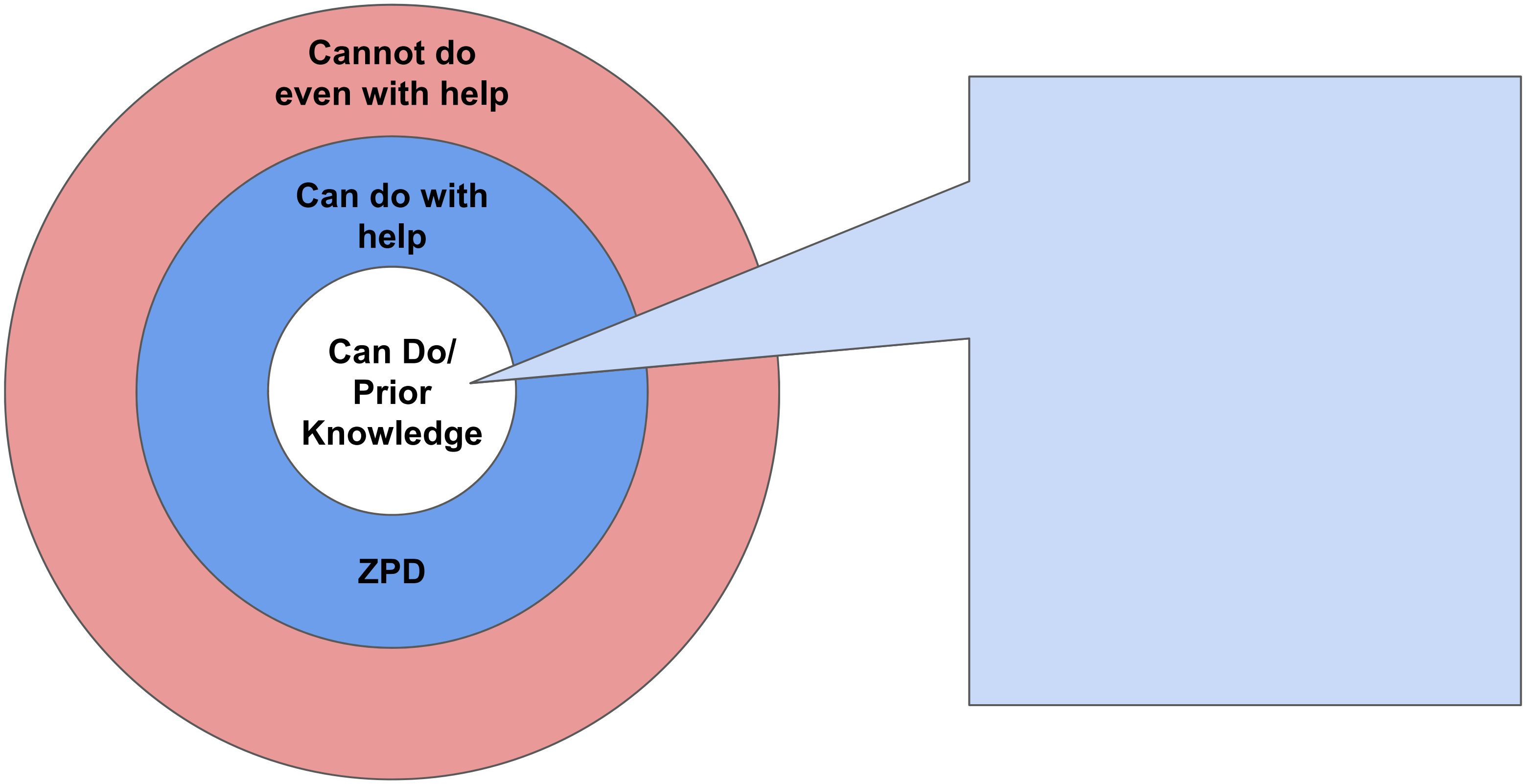

- **Learning objective:**
    - We will **define** the “zone of proximal development,” and **explain** how it is useful in choosing a level of complexity for our LIs

- **Performance Target**

	0	1	2
**Complexity**	The entire lesson is either so complex that it is inaccessible, or so simple that it offers no new knowledge	Some portion of the lesson is either so complex that it is inaccessible, or so simple that it offers no new knowledge	The lesson is complex yet accessible, and builds upon the group's prior knowledge

Practice: You're asked to deliver a lesson on diabetic neuropathy in 3 different contexts. For each of the scenarios below, use **context clues** to decide what prior knowledge you might count on your learner having as you determine how complex to make your lesson?

- You're in week 2 of the basic sciences block and your PBL group cannot decide exactly what "Diabetic neuropathy" means, this become a learning issue.
- A patient asks you why their type 2 diabetes has caused numbness in their feet. They currently work as an engineer.
- At home, retired relative asks you why their type 2 diabetes has caused numbness in their feet. They used to work as an accountant.

- **Possible** answer:
    - **You're in week 2 of the basic sciences block and your PBL group cannot decide exactly what "Diabetic neuropathy" means, this become a learning issue.**
    - We can likely count on college level biology and biochemistry, as well as some recent review of biochemical pathways during IMS.

- **Possible** answer:
  - **A patient asks you why their type 2 diabetes has caused numbness in their feet. They currently work as an engineer.**
  - We can count on college and or graduate level understanding of math, physics, and science generally. Uncertain how much specific biology or physiology knowledge may be present. You could consider a quick assessment of prior knowledge such as asking: “What do you know about your condition right now?”

- **Possible** answer:
    - **At home, retired relative asks you why their type 2 diabetes has caused numbness in their feet. They used to work as an accountant.**
    - We can count on a general comfort math and reasoning, and some distant high school level biological sciences. Beyond that, we could assume this person has little current knowledge of biology or physiology. Uncertain how much specific biology or physiology knowledge may be present. You should consider a quick assessment of prior knowledge such as asking: “What do you know about your condition right now?”
