# Supplementary material for: Teaching Skills Training for Pre-clinical Medical Students Through Weekly Problem-Based Learning Teaching Topic Presentations and Directed Feedback
Source: Med Sci Educ. 2023 Oct 18;33(6):1473–80. doi: 10.1007/s40670-023-01912-x (PMC10767181; doi:10.1007/s40670-023-01912-x)
Supplement: Supplementary file 4 — Supplementary file4 (PDF 190 KB) [file 40670_2023_1912_MOESM4_ESM.pdf]

KIRK KERKORIAN
SCHOOL OF MEDICINE | UNLV

Teaching Strategies for Effective LIs

❖ Engagement

Greg Schreck M.D. M.Ed. Rosalie Kalili M.D.

What was the last thing you read?

- **Learning objective:**
  - We will **define** “engagement” as an educational term, and **explore** how we can actively engage learners in our LIs

What does it mean to “engage” a learner?

Low vs high levels of engagement

So how do we make LIs engaging?

Webb's Depth of Knowledge levels:

Webb's Depth of Knowledge levels:

Level One

Recall

List 3 gram positive bacteria

Fill in the blank

Webb's Depth of Knowledge levels:

Level One	Level Two
Recall	Skills and Concepts
List 3 gram positive bacteria<br><br>Fill in the blank	Answer a multiple choice question<br><br>Perform an H&P

Webb's Depth of Knowledge levels:

Level One	Level Two	Level Three
Recall	Skills and Concepts	Strategic Thinking
List 3 gram positive bacteria<br><br>Fill in the blank	Answer a multiple choice question<br><br>Perform an H&P	Answer an open ended question<br><br>select tests to differentiate diagnoses

Webb's Depth of Knowledge levels:

Level One	Level Two	Level Three	Level Four
Recall	Skills and Concepts	Strategic Thinking	Extended Thinking
List 3 gram positive bacteria<br><br>Fill in the blank	Answer a multiple choice question<br><br>Perform an H&P	Answer an open ended question<br><br>select tests to differentiate diagnoses	Integrate history, exam, Labs, and imaging, to formulate an assessment and plan

Webb's Depth of Knowledge levels:

Level One	Level Two	Level Three	Level Four
Recall	Skills and Concepts	Strategic Thinking	Extended Thinking
Jeopardy!	Deal or No Deal	Family Feud	Survivor

Webb’s Depth of Knowledge levels:

Level One	Level Two	Level Three	Level Four
Recall	Skills and Concepts	Strategic Thinking	Extended Thinking
List 3 gram positive bacteria<br><br>Fill in the blank	Answer a multiple choice question<br><br>Perform an H&P	Answer an open ended question<br><br>select tests to differentiate diagnoses	Integrate history, exam, Labs, and imaging, to formulate an assessment and plan

So can we just talk for 5 straight minutes and then ask a multiple choice question?

Webb's Depth of Knowledge levels:

Level One	Level Two	Level Three	Level Four
Recall	Skills and Concepts	Strategic Thinking	Extended Thinking
List 3 gram positive bacteria<br><br>Fill in the blank	Answer a multiple choice question<br><br>Perform an H&P	Answer an open ended question<br><br>select tests to differentiate diagnoses	Integrate history, exam, Labs, and imaging, to formulate an assessment and plan

Engagement = Durable understanding

- **Learning objective:**
    - We will **define** “engagement” as an educational term, and **explore** how we can actively engage learners in our LIs

- **Performance Target**

	0	1	2
Engagement	Lesson provides no opportunities for active student engagement	Lesson provides opportunities for students to recall or list information	Lesson provides multiple opportunities for student engagement, AND some of these opportunities go beyond simply recalling or listing information

Practice: Rank the following activities from least engaging to most engaging (i.e. lowest to highest Webb's DoK level)

A. Read a patient vignette and compare DDx of meningitis vs migraine
B. Recall as many symptoms of hypoglycemia as you can
C. Create a vignette of a patient experiencing a silent MI
D. Infer the answer to a multiple choice question
E. Assess a patient vignette and formulate a differential diagnosis
F. Perform a complete patient H&P
G. List 3 antibiotics effective against staph aureus

- **Possible** answer:
    - **G, B, D, F, A, E, C**
        - This may not be the only “right answer”
    - **Bonus:** what do you think is the Webb’s DoK level of the activity you just did?
