# Supplementary material for: Teaching Skills Training for Pre-clinical Medical Students Through Weekly Problem-Based Learning Teaching Topic Presentations and Directed Feedback
Source: Med Sci Educ. 2023 Oct 18;33(6):1473–80. doi: 10.1007/s40670-023-01912-x (PMC10767181; doi:10.1007/s40670-023-01912-x)
Supplement: Supplementary file 5 — Supplementary file5 (PDF 181 KB) [file 40670_2023_1912_MOESM5_ESM.pdf]

KIRK KERKORIAN SCHOOL OF MEDICINE | UNLV

Teaching Strategies for Effective LIs

❖ Relevance and Resources

Greg Schreck M.D. M.Ed. Rosalie Kalili M.D.

Test Day

- **Learning objectives:**
  - We will **explain** why it is important to form connections between our LI and the real world
  - We will **explain** why it is important to use reliable and accessible resources for LI creation

Why does PBL work?

The power of connections

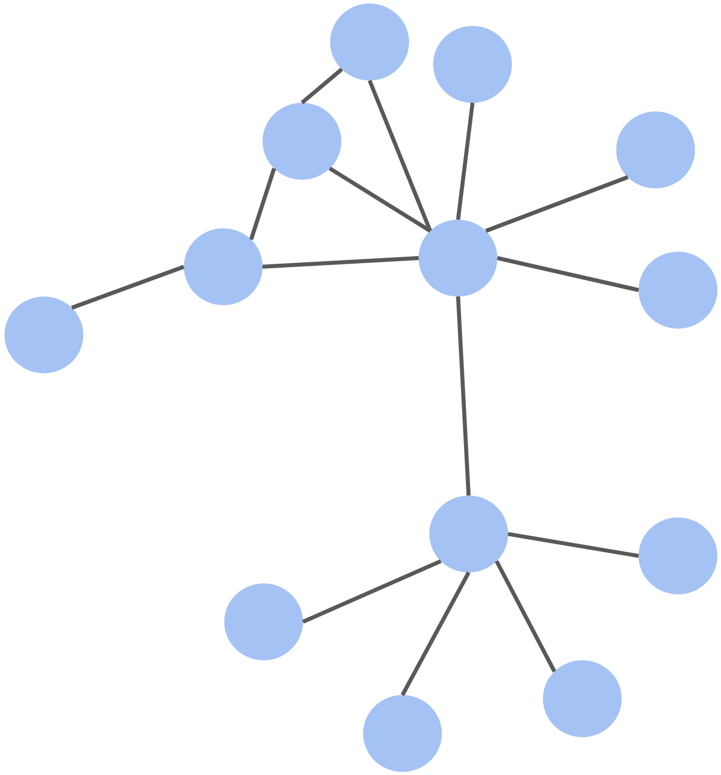

The power of connections

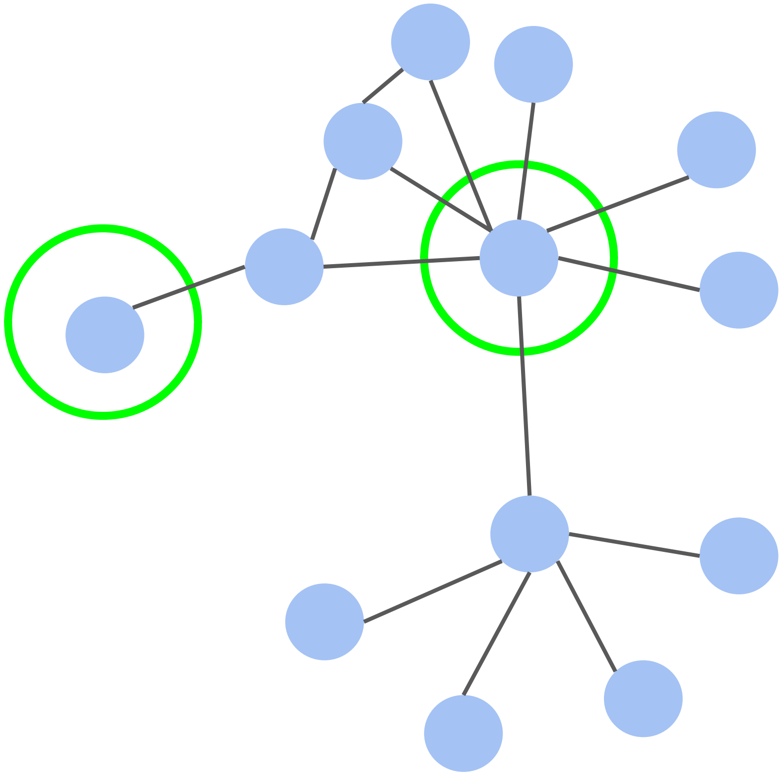

How else can we help our audience remember our LI?

The power of review

How do we facilitate review?

Resources

- **Healthcare professional associations**
  - ACS, ACOG, AAFP, etc.
- **Government health organizations**
  - CDC, WHO, NIH
- **Course Textbooks**
  - Robbins, Costanzo, Harrisons
- **Point of care tools**
  - UpToDate, Dynamed, choosing wisely
- **Peer reviewed Journals**

Webb's Depth of Knowledge levels:

Level One	Level Two	Level Three	Level Four
Recall	Skills and Concepts	Strategic Thinking	Extended Thinking
List 3 gram positive bacteria<br><br>Fill in the blank	Answer a multiple choice question<br><br>Perform an H&P	Answer an open ended question<br><br>select tests to differentiate diagnoses	Integrate history, exam, Labs, and imaging, to formulate an assessment and plan

- **Learning objectives:**
  - We will **explain** why it is important to form connections between our LI and the real world
  - We will **explain** why it is important to use reliable and accessible resources for LI creation

- **Performance Targets**

	0	1	2
**Relevance**	Lesson is not related to the patient case	Lesson content relates to the patient case but is not explicitly connected	Presenter makes explicit connection between lesson content and patient case
**Resources**	No resources cited	Credible resources are cited	Credible resources beyond USMLE prep resources are cited

Practice:

- Find the “LI Rubric” in the resource drive for this course
  - That is your reference to review the teaching strategies for effective LIs covered in this mini series
  - Assess your own LIs helps to help you build a **connection** between these teaching strategies and the real world, and to **engage** with these strategies to facilitate a deep and durable understanding!

We hope you learned something!

Thanks for watching, and remember that we made this course because we believe that if you focus on these key areas, you will grow as a teacher and communicator of medical information; skills that will help you as a doctor. We also hope having these strategies will grant some direction and lighten the load of creating your own lessons from scratch. Good luck!
